# The Impact of Psychosocial Factors on the Human—Pet Bond: Insights from Cat and Dog Owners

**DOI:** 10.3390/ani15131895

**Published:** 2025-06-26

**Authors:** Garikoitz Azkona

**Affiliations:** Department of Basic Psychological Processes and Their Development, Euskal Herriko Unibertsitatea (UPV/EHU), Tolosa Hiribidea 70, 20018 Donostia, Spain; garikoitz.azkona@ehu.eus

**Keywords:** cat, dog, pet owner, social support, loneliness, mental well-being, attachment, person substitution

## Abstract

This study underscores how perceived social support, loneliness, and mental well-being shape the emotional bonds that Spanish cat and dog owners form with their pets. A total of 298 participants completed an online survey, including 109 cat owners (36.5%) and 189 dog owners (63.4%). Standardized and validated instruments were used: the Medical Outcomes Study Social Support Survey (MOS-SSS), the UCLA Loneliness Scale, the Warwick–Edinburgh Mental Well-Being Scale (WEMWBS), and the Lexington Attachment to Pets Scale (LAPS). The results confirmed that the attachment to dogs tends to be stronger than to cats, with demographic factors such as gender and age also playing a significant role in the attachment levels. While psychosocial variables did not directly predict the overall attachment scores, lower perceived social support was significantly associated with higher scores on the person substitution subscale among dog owners, indicating that dogs may serve as emotional substitutes for human companionship in socially vulnerable individuals. No comparable association was found among cat owners. These findings highlight species-specific differences in the human–pet bond and emphasize the multifaceted nature of attachment. This study underscores the importance of considering both demographic and psychosocial factors to better understand how pets contribute to human emotional well-being.

## Simple Summary

This study explored the factors influencing attachment to cats and dogs, focusing on demographic and psychosocial variables in the Spanish population. Consistent with previous research, the attachment to dogs was generally stronger than to cats, with gender and age significantly affecting the attachment levels—women and younger owners reported higher attachment. Psychosocial factors such as social support, loneliness, and mental well-being did not directly predict overall attachment but influenced specific emotional dimensions, particularly the person substitution (PS) aspect in dog owners. This suggests that individuals with lower social support may form compensatory bonds with their dogs, viewing them as substitutes for human companionship. No such clear pattern was observed for cat owners, reflecting species-specific differences in attachment styles. Dogs appear to play a crucial role in mitigating loneliness and providing emotional support, while cats offer a different, perhaps less intense but still meaningful, form of companionship. This study highlights the complex, multifaceted nature of human–animal bonds and underscores the importance of considering species differences when examining these relationships.

## 1. Introduction

In Spain, cats and dogs are the most popular and preferred pets, reflecting their prominent role in many households across the country [1,2]. An estimated 28 million cats and dogs live in Spain, with more than 40% of households having at least one of these pets [3]. Over the course of human evolution, the nature of our relationship with these animals has undergone a significant transformation, shifting from primarily utilitarian roles, such as hunting assistance, protection, or pest control, to being perceived as valued companions. Today, cats and dogs are often regarded not just as pets but as integral members of the family, fulfilling social and emotional roles comparable to those of close friends or relatives [4,5]. This profound change mirrors a fundamental human trait: the innate need to form affiliative bonds and seek social connections [6,7].

Several theoretical frameworks have been proposed to explain the origins and persistence of these bonds between humans and animals. One of the most widely recognized perspectives is the biophilia hypothesis, which suggests that humans possess an inherent affinity for nature and living beings, including animals. This intrinsic connection is thought to have evolutionary roots, promoting well-being and survival through interactions with the natural environment [8,9]. Complementing this, attachment theory, originally developed to explain the bonds between humans, particularly between children and caregivers, has been extended to include human–animal relationships. According to this model, the formation of emotional bonds with animals is a natural extension of our broader social tendencies, allowing for comfort, security, and emotional support similar to that provided by human relationships [10,11]. Together, these theories help elucidate why companion animals play such a central role in human lives and why the affective connections between humans and their pets are often so deep and enduring.

Research shows that attachment to pets differs markedly by species, with dog owners generally reporting stronger bonds than cat owners [12,13,14,15,16,17,18]. This variation may be attributed to the distinct behavioral traits and social tendencies exhibited by dogs and cats in their interactions with humans. Dogs are typically considered more trainable, interactive, and attuned to human cues, often seeking and responding actively to social engagement. Their loyal and expressive nature facilitates frequent and meaningful interactions, which can foster deeper emotional connections with their owners [19,20]. In contrast, cats are often perceived as more independent and self-sufficient animals, displaying less overt social dependence and a more selective approach to interaction. This relative autonomy may result in fewer opportunities for intense or frequent bonding experiences, potentially leading to generally less intense attachment levels among cat owners [20,21]. Understanding these species-specific differences is crucial to interpreting variations in the human–animal bond.

Additionally, various demographic, social, and psychological characteristics of pet owners have been identified as important factors influencing the strength and quality of the human–pet bond. These owner-specific variables include gender, age, educational level, residential area (urban versus rural), income range, relationship status (such as single or partnered), and the size and composition of their social networks [12,13,14,15,17,18,22,23,24,25,26,27,28,29,30,31]. Furthermore, studies have shown that attachment levels can also be associated with specific personality traits of pet owners, such as extraversion, neuroticism, and openness to experience, which have been linked to variations in levels of attachment to pets [27,32,33]. Each of these factors influences how individuals relate to their pets, highlighting the complex interplay of owner characteristics that shape the human–pet bond.

The connection between social support, loneliness, and mental well-being is well-documented. Research shows that perceived loneliness is a strong predictor of mental health outcomes, while social support plays a partial mediating role in this relationship, helping to buffer the negative impact of loneliness on mental well-being [34,35,36]. One widely accepted explanation for the benefits derived from relationships with companion animals, such as cats and dogs, is that pet ownership can enhance social support and reduce feelings of loneliness [21,37,38,39,40,41]. Moreover, extensive research has demonstrated the positive effects of human–animal interactions on health outcomes, particularly in reducing stress and promoting emotional well-being [42,43,44,45,46,47]. However, some studies suggest that there may be no significant differences in loneliness between pet owners and non-owners living alone, and that certain pet owners may experience greater loneliness and psychological distress [48,49]. Additionally, a recent systematic review concluded that attachment to pets is not always directly associated with improvements in mental health and well-being [50].

This article aims to address the lack of research in Spain on the impact of psychosocial factors, such as perceived social support, loneliness, and mental well-being, on the degree of emotional attachment people develop toward their dogs or cats. To explore this relationship, we employ widely used and validated instruments, including the Medical Outcomes Study Social Support Survey (MOS-SSS), the University of California, Los Angeles (UCLA) Loneliness Scale, the Warwick–Edinburgh Mental Well-Being Scale (WEMWBS), and the Lexington Attachment to Pets Scale (LAPS).

## 2. Materials and Methods

### 2.1. Participants and Procedure

Between September 2024 and April 2025, participant recruitment was carried out online using a snowball sampling method. Initial respondents were invited to refer other cat or dog owners to complete the survey. The recruitment targeted individuals residing in Spain, aged 18 or older, who owned either a cat or a dog at the time they completed the survey.

A cover letter accompanied the questionnaire, informing participants that the data collected would be used exclusively for scientific purposes and that all the responses would remain fully anonymous. All the participants voluntarily provided informed consent before completing the online questionnaire via the Google Drive platform. To ensure a species-specific assessment of the human–animal bond and to minimize the potential bias stemming from multi-pet ownership, participants with more than one pet were instructed to complete the survey based on the animal with whom they felt the closest bond.

This study followed the ethical principles outlined in the Declaration of Helsinki. All the procedures and consent protocols received approval from the Ethics Committee for Human-Related Research (CEISH) at the University of the Basque Country (UPV/EHU), 2023/159.

### 2.2. Instruments

This study collected demographic information such as gender, age (in years), area of residence (rural or urban), household composition (whether living alone or not), romantic relationship status (whether currently in a romantic relationship), and number of individuals in the immediate social circle.

Social support was evaluated using the Spanish version of the MOS-SSS (α = 0.94). This 19-item scale uses a 5-point Likert response format (1 = never to 5 = always). Based on the total scores, social support was categorized as low (≤38), average (39–57), or high (≥58) [51]. To assess loneliness, the Spanish version of the UCLA Loneliness Scale was utilized (α = 0.95). This instrument includes 10 items rated on a 4-point Likert scale (1 = never to 4 = often), with the resulting scores classified as low (<20), average (20–30), or high (>30) [34]. Subjective mental well-being was assessed using the Spanish version of the WEMWBS (α = 0.93), which comprises 14 items rated on a 5-point Likert scale (1 = never to 5 = always). The scores were grouped into low (≤40), average (41–58), or high (≥59) [52].

Regarding human–animal relationships, participants first identified the species (dog or cat) of the pet considered when responding to the survey. Then, participants had to respond to the Spanish version of the LAPS (α = 0.89) [17]. This 23-item scale uses a four-point Likert response format (0 = strongly disagree to 3 = strongly agree) and includes three subscales: general attachment (GA), reflecting emotional closeness to the pet (items 10, 11, 12, 13, 15, 17, 18, 19, 21R, and 22); person substitution (PS), measuring the pet’s emotional role in the owner’s life (items 1, 2, 3, 4, 5, 6, 7 and 9); and animal rights (AR), assessing the perceived moral status and role of the pet within the household (items 3, 8R, 14, 16 and 20).

### 2.3. Statistical Data Analysis

The statistical analysis used jamovi (version 2.3.21.0) and GraphPad Prism (version 10.3.1), with the significance threshold set at *p* < 0.05. Descriptive statistics—frequency (%), mean, standard deviation (SD), median, and range—summarized the sample characteristics. The Shapiro–Wilk test checked for normality and indicated a non-parametric distribution for all the variables. Due to the binary nature of all the variables, the Mann–Whitney U test was used to identify differences in the scale scores. Rank biserial correlation (rrb) was used to compute the effect sizes, interpreted as <0.3 (small), 0.3–0.5 (moderate), and >0.5 (large). The bivariate Spearman’s rho was used to examine the correlations between variables, with interpretation cutoffs of <0.19 (small), 0.20–0.29 (moderate), and >0.30 (large) [53]. Linear regression analyses were performed to determine the independent influences of the demographic (gender, age, living area, living alone, and romantic relationship) and psychosocial variables (social support, loneliness, and mental well-being).

## 3. Results

### 3.1. General Information

In total, 298 participants completed the survey: 109 cat owners (36.5%) and 189 dog owners (63.4%) (Table 1). The majority of participants identified as women (*n =* 225, 75.5%), lived in urban areas (*n =* 223, 74.8%), did not live alone (*n =* 253, 84.9%), and were in a romantic relationship (*n =* 165, 55.3%). Their ages ranged from 18 to 75 years (mean: 39.5, SD: 13.9; median: 38). On average, participants had 10 people in their closest social circle (SD: 5.61, median: 9, range: 1–30). The vast majority of participants reported high levels of perceived social support (*n =* 265, 88.9%), low to average levels of perceived loneliness (*n =* 283, 94.9%), and average to high levels of mental well-being (*n =* 282, 94.6%). The cat and dog owners did not differ significantly in the number of people in their closest social circle, perceived social support, levels of loneliness, or mental well-being scores (Supplementary Table S1). The analysis revealed significant correlations among the psychosocial variables examined (Supplementary Table S2).

### 3.2. Attachment to Pets

The dog owners scored significantly higher than the cat owners across all the dimensions of the Lexington Attachment to Pets Scale (Table 2).

**Figure 1:**
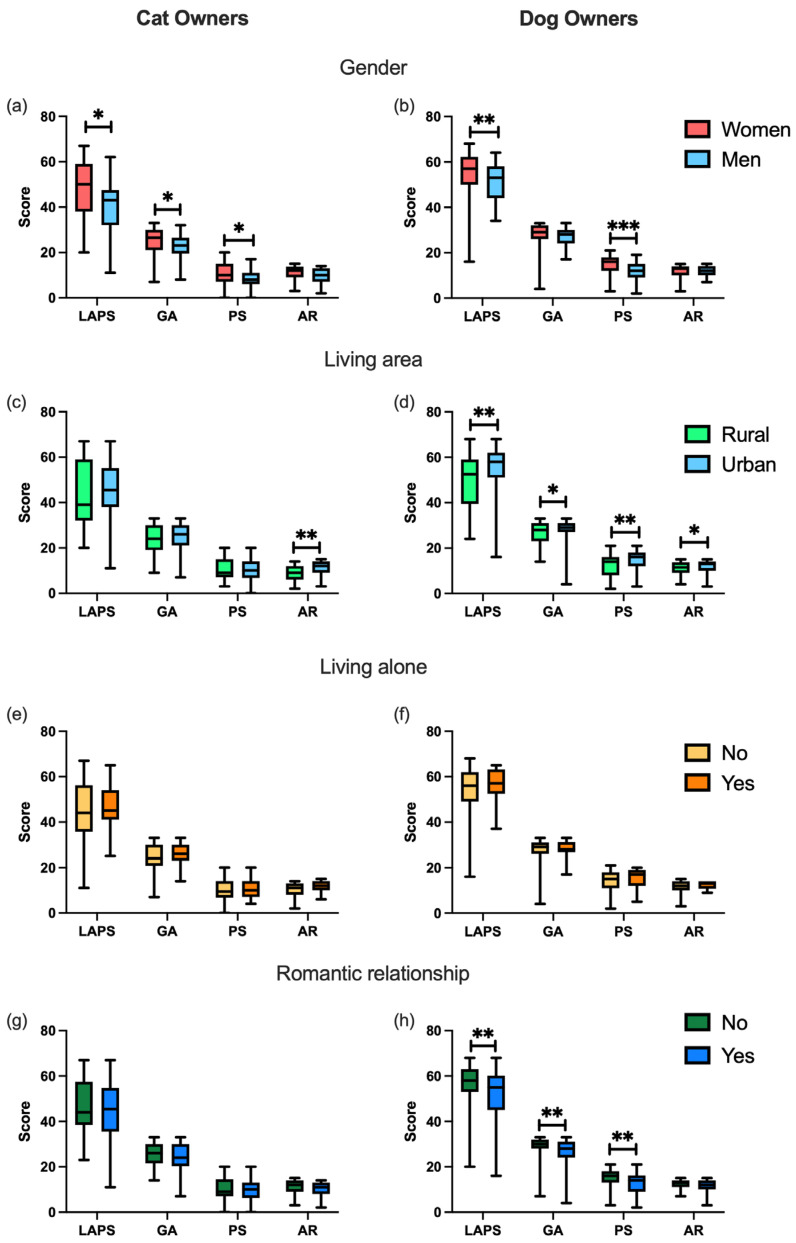
Lexington Attachment to Pets Scale (LAPS) results, including the general attachment (GA), person substitution (PS), and animal rights (AR) subscales, by (**a**,**b**) gender, (**c**,**d**) living area, (**e**,**f**) living alone, and (**g**,**h**) romantic relationship status, separately by species. Data are presented as group medians (min–max). * *p* < 0.05, ** *p* < 0.01, *** *p* < 0.001.

Given these differences, we conducted the remaining exploratory analyses separately for the cat and dog owners. Additional analyses revealed that female cat owners scored significantly higher than male cat owners on the total LAPS (U = 868, *p* = 0.011, rrb = 0.304) and the GA (U = 870, *p* = 0.011, rrb = 0.307) and PS (U = 977, *p* = 0.026, rrb = 0.269) subscales (Figure 1a). Similarly, female dog owners scored higher on the LAPS (U = 2061, *p* = 0.004, rrb = 0.296) and the PS (U = 1812, *p* < 0.001, rrb = 0.381) subscale (Figure 1b). Among the cat owners, those living in rural areas scored lower on the AR subscale than those living in urban areas (U = 628, *p* = 0.007, rrb = 0.365). Among the dog owners, those living in urban areas scored higher across all the LAPS dimensions: total LAPS (U = 2620, *p* = 0.005, rrb = 0.264), GA (U = 2904, *p* = 0.049, rrb = 0.185), PS (U = 2603, *p* = 0.004, rrb = 0.269) and AR (U = 2836, *p* = 0.028, rrb = 0.204) (Figure 1d). The analysis revealed no significant differences in the LAPS scores according to household composition for either the cat or dog owners (Figure 1e,f). Being in a romantic relationship did not influence attachment among the cat owners (Figure 1g). However, the dog owners in a romantic relationship scored significantly lower on the total LAPS (U = 3314, *p* = 0.005, rrb = 0.240) and the GA (U = 3377, *p* = 0.008, rrb = 0.225), and PS (U = 3272, *p* = 0.003, rrb = 0.249) subscales (Figure 1h).

Correlation analysis revealed a large negative correlation between the cat owners’ age and the total LAPS score (−0.381, *p* < 0.001), as well as the GA (−0.303, *p* < 0.001), PS (−0.321, *p* < 0.001), and AR (−0.428, *p* < 0.001) subscales. Likewise, the dog owners’ age was negatively correlated with the LAPS (−0.285, *p* < 0.001), GA (−0.283, *p* = 0.005), PS (−0.341, *p* < 0.001), and AR (−0.179, *p* = 0.014). Among the cat owners, no correlation was found between the LAPS scale and its subscales and the number of people in their intimate social circles. Among the dog owners, only a weak negative correlation was observed between the number of close people and the PS subscale (−0.168, *p* = 0.021).

In summary, the exploratory analyses revealed that dog owners reported significantly higher levels of attachment to their pets than cat owners across all the dimensions of the LAPS. Additionally, women showed greater attachment than men among both the cat and dog owners. Urban pet owners, particularly those with dogs, demonstrated stronger attachment scores compared to rural owners. Household composition did not influence the attachment levels, but being in a romantic relationship was associated with lower attachment scores among the dog owners. Correlation analyses further showed a significant negative relationship between age and attachment in both groups, which was especially pronounced among the cat owners. The dog owners also showed negative correlations between age and attachment, though these were generally weaker. Finally, while the number of close social contacts did not relate to attachment in the cat owners, the dog owners exhibited a weak negative correlation between the size of their intimate social circle and their emotional reliance on their pet. Overall, these findings suggest that demographic factors, including gender, residential area, age, and romantic involvement, may differentially affect the strength of the human–pet bond depending on the species.

### 3.3. Psychosocial Factors and Pet Attachment

Following the initial exploratory analyses, we aimed to investigate the potential relationships between psychosocial factors, social support, loneliness, and mental well-being, and the dimensions of the LAPS (Table 3). For the cat owners, no statistically significant correlations emerged between the psychosocial variables and the total LAPS score or its subscales. In contrast, among the dog owners, significant correlations were primarily observed with the person substitution subscale. Specifically, social support and mental well-being showed a moderate negative correlation with PS (*p* = 0.001), while loneliness showed a moderate positive correlation with PS (*p* = 0.003). Additionally, the total LAPS score for the dog owners was weakly negatively correlated with social support (*p* = 0.038) and mental well-being (*p* = 0.017), and weakly positively correlated with loneliness (*p* = 0.043). These results suggest that among dog owners, attachment, particularly the emotional reliance on the pet as a substitute for a person, relates to psychosocial well-being, whereas this pattern does not appear among cat owners.

To further investigate the relationships between psychosocial factors and attachment to pets among the dog owners, we conducted multiple regression analyses. These analyses aimed to evaluate the independent effects of psychosocial factors on the total LAPS and PS scores, while controlling for demographic variables. By focusing exclusively on the dog owners, where the preliminary analyses indicated significant associations, this approach provides a clearer understanding of the key predictors of attachment in this group (Table 4). For the LAPS total score, the linear regression model explained approximately 17.8% of the variance in the LAPS scores (R^2^ = 0.178; _adj_R^2^ = 0.141; F_(8, 179)_ = 4.85, *p* < 0.001). Among the predictors, age and living area had significant effects on the LAPS score, indicating that being young and living in an urban area were associated with higher levels of attachment to their dog. For the PS subscale, the model accounted for 28.2% of the variance (R^2^ = 0.282; _adj_R^2^ = 0.250; F_(8, 179)_ = 8.79, *p* < 0.001). Significant predictors included gender, age, living area, and social support. These results suggest that being female, younger, living in an urban area, and having perceived low social support were associated with higher PS scores.

In summary, the regression analyses indicated that age and living area were significant predictors of both the LAPS and PS scores, suggesting that younger individuals and those in urban living environments reported stronger emotional bonds with their dogs and a greater tendency to view them as substitutes for human relationships. Additionally, gender significantly predicted person substitution, with women showing a higher tendency toward this behavior, and lower social support was also associated with increased person substitution.

## 4. Discussion

This study investigated how psychosocial factors relate to attachment to pets, focusing on potential differences between dog and cat owners. Our findings highlight important distinctions in the ways social support, loneliness, and mental well-being correlate with attachment dimensions in these groups.

An initial exploratory analysis assessed the impact of demographic factors on attachment levels. Consistent with previous research conducted across diverse populations, our findings suggest that dog owners generally report stronger attachments than cat owners, and women tend to report stronger attachment bonds than men [12,13,14,15,16,17,18,22,23,26,27,28,29,30,31,32,54]. When analyzing demographic factors by pet species, we observed that age, living area, and romantic relationship status significantly influenced the degree of attachment, but only in dog owners. Specifically, younger participants exhibited higher levels of attachment to their pets compared to older participants, a pattern aligned with recent studies [14,27,30]. Additionally, dog owners living in urban areas obtained higher LAPS scores and subscale scores, suggesting that the area of residence plays a role in pet attachment [23,24,25]. Furthermore, relationship status appears to affect attachment, as individuals with stronger attachments to their dogs often displayed weaker emotional bonds with other humans [15,23].

Overall, the results of the present study are consistent with prior studies reporting high levels of social support and moderately low levels of loneliness in Spain [34,51,55,56,57,58,59,60,61,62]. The lack of differences in the perceived social support, loneliness, and mental well-being between cat owners and dog owners aligns with studies suggesting that pet ownership may have similar effects on these psychosocial factors regardless of the type of animal [38,41,63,64,65,66]. The absence of a non-pet-owning control group limits the conclusions about the overall effects of pet ownership on these psychosocial variables.

Among the dog owners, the analyses revealed a modest but significant correlation between attachment and psychosocial factors. These results suggest that dog owners who experience lower social support and/or mental well-being and higher loneliness tend to develop stronger attachments to their pets. This pattern was especially pronounced in terms of the person substitution subscale, indicating that dog owners with limited emotional or social resources may increasingly rely on their pets to fill gaps in their human relationships, consistent with the concept of ‘person substitution’ described in the prior literature [9,38,39]. This compensatory attachment pattern is consistent with dogs’ sociable and interactive natures, which often encourages emotional bonding and facilitates social connection [67,68,69]. In contrast, the cat owners did not exhibit significant associations between attachment measures and psychosocial factors. This lack of correlation may stem from the different behavioral traits and social dynamics characteristic of cats. Known for their autonomy and relative independence, cats may foster attachments that are less contingent on their owners’ social support or emotional needs. The human–cat bond may thus represent a distinct form of companionship, less oriented toward compensatory social support and more characterized by coexistent or complementary relationships [70,71,72]. However, this pattern might also be culturally influenced, as a recent study conducted in China found a positive relationship between attachment level and social support among cat owners [73].

This study has several limitations that may affect the interpretation and generalizability of the findings. First, the data were collected through self-report questionnaires, which can be subject to biases such as social desirability and inaccuracies in participants’ recollections. Second, the sample is not fully representative of the general population, as there was a notable overrepresentation of women. This gender imbalance may obscure potential differences in pet attachment based on sex, as previous research has shown gender to be a relevant factor in attachment patterns. Third, most participants in the sample reported high levels of mental well-being, strong perceived social support, and low levels of loneliness. This homogeneity may limit the ability to detect the supportive effects of pets, which tend to be more pronounced in individuals experiencing social exclusion or elevated stress levels, as suggested by earlier studies [74,75,76,77]. Fourth, this study focused exclusively on the strength of the owner–pet bond. Future research is recommended to explore this area further, placing greater emphasis on both the strength and the quality of the bond, including different attachment styles. Finally, the cross-sectional design of this study prevents any conclusions being drawn about the causality between the variables examined. Future research should adopt longitudinal designs and include more diverse samples to better understand the potential impact of pets on mental health across different contexts and populations. It would also be important to include a comparison group of non-pet-owners, which was not part of the current study, in order to more clearly isolate the effects of pet ownership. This could be a valuable direction for future investigations.

## 5. Conclusions

This study confirms that attachment to dogs is generally stronger than attachment to cats and that owner demographic factors, such as gender and age, play a significant role in shaping these bonds. While the perceived social support, loneliness, and mental well-being may not directly influence the overall strength of attachment, they do influence the emotional dynamics within the human–dog relationship, especially through the mechanism of person substitution. These findings emphasize the complexity and multifaceted nature of human–animal bonds, highlighting distinct species-specific pathways through which humans develop meaningful attachments to their pets. Understanding these differences is essential for appreciating the diverse roles pets play in human emotional well-being.

## Figures and Tables

**Table 1:** General information by pet ownership.

	Cat Owner	Dog Owner
	n (%)	
Gender		
Female	75 (68.8%)	150 (79.4%)
Male	34 (31.2%)	39 (20.6%)
Living area		
Rural	23 (21.1%)	52 (27.5%)
Urban	86 (78.9%)	137 (72.5%)
Living alone		
No	86 (78.9%)	167 (88.4%)
Yes	23 (21.1%)	22 (11.6%)
Romantic relationship		
No	49 (44.9%)	83 (43.9%)
Yes	60 (55.1%)	105 (56.1%)
Social support		
Low	6 (5.5%)	1 (0.5%)
Average	9 (8.6%)	17 (8.9%)
High	94 (85.9%)	171 (90.6%)
Loneliness		
Low	48 (44%)	96 (50.7%)
Average	51 (46.8%)	88 (46.6%)
High	10 (9.2%)	5 (2.7%)
Mental well-being		
Low	8 (7.3%)	8 (4.2%)
Average	51 (46.8%)	85 (44.9%)
High	50 (45.9%)	96 (50.9%)

**Table 2:** LAPS score by pet ownership. ** *p* < 0.01 and *** *p* < 0.001.

	Group	Mean	SD	Median	Range	U	*p*	rbb
LAPS	Cat owner	45.4	12.35	44	11–67	6153	<0.001 ***	0.403
	Dog owner	53.8	10.49	56	16–68			
GA	Cat owner	24.7	5.94	25	7–33	6799	<0.001 ***	0.340
	Dog owner	27.9	4.77	29	4–33			
PS	Cat owner	10.2	4.78	10	0–20	5759	<0.001 ***	0.441
	Dog owner	14.1	4.70	15	2–21			
AR	Cat owner	10.6	3.23	11	2–15	8042	0.001 **	0.219
	Dog owner	11.8	3.23	12	3–15			

**Table 3:** Correlation matrix between social support, loneliness, mental well-being, and the LAPS scores by animal species in owners. * *p* < 0.05, ** *p* < 0.01.

		Social Support	Loneliness	Mental Well-Being
LAPS	Cat owners	0.087	0.096	−0.087
	Dog owners	−0.151 *	0.147 *	−0.173 *
GA	Cat owners	0.117	0.015	−0.014
	Dog owners	−0.026	0.046	−0.080
PS	Cat owners	−0.022	0.183	−0.145
	Dog owners	−0.236 **	0.215 **	−0.234 **
AR	Cat owners	0.163	0.099	−0.048
	Dog owners	−0.104	0.125	−0.143

**Table 4:** Results of the linear regression analysis for the total LAPS and the PS subscale involving predictor variables. * *p* < 0.05, ** *p* < 0.01, *** *p* < 0.001. Reference level: woman, urban, living alone, no romantic relationship.

		Standardized β	95% CI	t	*p*
LAPS					
	Gender	−0.2919	−0.638–0.0538	−1.666	0.097
	Age	−0.2593	−0.415–−0.1034	−3.282	0.001 **
	Living area	−0.4184	−0.726–−0.1113	−2.688	0.008 **
	Living alone	−0.1670	−0.611–0.2766	−0.743	0.459
	Romantic relationship	−0.1273	−0.439–0.1842	−0.806	0.421
	Social support	−0.1127	−0.297–0.0712	−1.209	0.228
	Loneliness	−0.0596	−0.255–0.1356	−0.602	0.548
	Mental well-being	−0.0933	−0.275–0.0882	−1.014	0.312
PS					
	Gender	−0.4882	−0.811–−0.1651	−2.982	0.003 **
	Age	−0.3154	−0.461–−0.1697	−4.271	<0.001 ***
	Living area	−0.4290	−0.716–−0.1419	−2.949	0.004 **
	Living alone	−0.1871	−0.602–0.2275	−0.890	0.374
	Romantic relationship	−0.0574	−0.349–0.2338	−0.389	0.698
	Social support	−0.1842	−0.356–−0.0124	−2.115	0.036 *
	Loneliness	−0.0195	−0.202–0.1630	−0.210	0.834
	Mental well-being	−0.0934	−0.263–0.0762	−1.087	0.279

## Data Availability

The study data are available in the Supplementary Table S3.

## Supplementary Materials

The following supporting information can be downloaded at https://www.mdpi.com/article/10.3390/ani15131895/s1, Table S1: Number of people in their closest social circle and social support, loneliness, and mental well-being scores between pet owners; Table S2: Correlation matrix between people in the inner social circle, social support, loneliness, and mental well-being; Table S3. Raw data from the study.

## Abbreviations

The following abbreviations are used in this manuscript:

AR	Animal Rights
GA	General Attachment
LAPS	Lexington Pet Attachment Scale
MOS-SSS	Medical Outcomes Study Social Support Survey
PS	Person Substitution
UCLA	University of California, Los Angeles
WEMWBS	Warwick–Edinburgh Mental Well-Being Scale
