# Supplementary material for: The Impact of Psychosocial Factors on the Human—Pet Bond: Insights from Cat and Dog Owners
Source: Animals (Basel). 2025 Jun 26;15(13):1895. doi: 10.3390/ani15131895 (PMC12248966; doi:10.3390/ani15131895)
Supplement: Supplementary file 1 [file animals-15-01895-s001.zip › animals-3715254 Supplementary Table S1 final.pdf]

animals

MDPI

Supplementary Table S1. Number of people in their closest social circle and social support, loneliness, and mental well-being scores between pet owners.

	Group	Mean	SD	Median	Range
**People in the closest social circle**	Cat owner	10.2	5.52	9	1-30
	Dog owner	9.9	5.79	9	1-30
**Social support**	Cat owner	75.3	18.4	79	26-95
	Dog owner	80.4	13.0	84	34-95
**Loneliness**	Cat owner	21.5	6.58	20	10-40
	Dog owner	19.8	5.24	19	10-34
**Mental well-being**	Cat owner	55.7	10.74	57	30-70
	Dog owner	57.8	8.96	58.5	21-70
