# Supplementary material for: The Impact of Psychosocial Factors on the Human—Pet Bond: Insights from Cat and Dog Owners
Source: Animals (Basel). 2025 Jun 26;15(13):1895. doi: 10.3390/ani15131895 (PMC12248966; doi:10.3390/ani15131895)
Supplement: Supplementary file 1 [file animals-15-01895-s001.zip › animals-3715254 Supplementary Table S2 final.pdf]

animals

Supplementary Table S2. Correlation matrix between people in the inner social circle, social support, loneliness, and mental well-being. n = 298; * p < 0.05, ** p < 0.01, *** p < 0.001.

	Inner circle	Social support	Loneliness	Mental well-being
Inner circle	—			
Social support	0.389 ***	—		
Loneliness	-0.276 ***	-0.567 ***	—	
Mental well-being	0.324 ***	0.620 ***	-0.592 ***	—
